# Rapid Succession of Actively Transcribing Denitrifier Populations in Agricultural Soil During an Anoxic Spell

**DOI:** 10.3389/fmicb.2018.03208

**Published:** 2019-01-08

**Authors:** Binbin Liu, Xiaojun Zhang, Lars R. Bakken, Lars Snipen, Åsa Frostegård

**Affiliations:** ^1^Faculty of Chemistry, Biotechnology and Food Science, Norwegian University of Life Sciences, Ås, Norway; ^2^State Key Laboratory of Microbial Metabolism and School of Life Sciences and Biotechnology, Shanghai Jiao Tong University, Shanghai, China

**Keywords:** denitrification, transcription, denitrifying genes, amplicon sequencing, OTU clustering

## Abstract

Denitrification allows sustained respiratory metabolism during periods of anoxia, an advantage in soils with frequent anoxic spells. However, the gains may be more than evened out by the energy cost of producing the denitrification machinery, particularly if the anoxic spell is short. This dilemma could explain the evolution of different regulatory phenotypes observed in model strains, such as sequential expression of the four denitrification genes needed for a complete reduction of nitrate to N_2_, or a “bet hedging” strategy where all four genes are expressed only in a fraction of the cells. In complex environments such strategies would translate into progressive onset of transcription by the members of the denitrifying community. We exposed soil microcosms to anoxia, sampled for amplicon sequencing of *napA/narG, nirK/nirS*, and *nosZ* genes and transcripts after 1, 2 and 4 h, and monitored the kinetics of NO, N_2_O, and N_2_. The cDNA libraries revealed a succession of transcribed genes from active denitrifier populations, which probably reflects various regulatory phenotypes in combination with cross-talks via intermediates (${\mathrm{NO}}_{2}^{-}$, NO) produced by the “early onset” denitrifying populations. This suggests that the regulatory strategies observed in individual isolates are also displayed in complex communities, and pinpoint the importance for successive sampling when identifying active key player organisms.

## Introduction

Denitrification in agricultural soils is recognized as a major source of nitric and nitrous oxide emissions to the atmosphere (Opdyke et al., 2009; Syakila and Kroeze, 2011; Butterbach-Bahl et al., 2013; Gerber et al., 2016). The process is a dissimilatory metabolism that enables facultative anaerobic microorganisms to respire nitrogen oxides during periods of anoxia. Complete denitrification is the reduction of nitrate (${\mathrm{NO}}_{3}^{-}$) to dinitrogen (N_2_) via nitrite (${\mathrm{NO}}_{2}^{-}$), nitric oxide (NO) and nitrous oxide (N_2_O). These four consecutive steps are catalyzed by the complex metallo-enzymes Nar, Nir, Nor, and N2OR (${\mathrm{NO}}_{3}^{-}$-, ${\mathrm{NO}}_{2}^{-}$-, NO-, and N_2_O reductase, encoded by *nar, nir, nor*, and *nos*, respectively). Nar reductases encompass the membrane bond NarG and the periplasmic NapA; Nir the copper containing NirK and the cytochrome cd_1_ NirS; and Nor the cytochrome c dependent cNor, the quinol dependent qNor and the less common copper-containing qCuANor (Shapleigh, 2013). The N2OR enzymes are separated into two main clades (I and II), both with two copper-containing redox centers (Hallin et al., 2017). Except for the two types of Nir, bacteria very seldom contain more than one type of the respective reductases (Graf et al., 2014). A complete denitrification pathway, comprising all four reduction steps of denitrification, is found among a wide range of taxonomically diverse bacteria, as well as in some Archaea (Shapleigh, 2013). In addition, there are many prokaryotes, and also some fungi, that have a truncated denitrification pathway where one or more of the steps are missing. This may be due either to a lack of the functional gene itself or another essential gene in the operon (Bergaust et al., 2008; Liu et al., 2013; Roco et al., 2017), or because transcriptional regulation or post-transcriptional mechanisms prevent or delay the expression of functional enzymes (Bergaust et al., 2010, 2012; Bueno et al., 2015). In a study by Chèneby et al. (2000) the numbers of denitrifiers (with or without N_2_O reduction), isolated from a range of soils, was 0.9–4.7% of all the aerobic/facultative anaerobic bacterial isolates. More recently, Lycus et al. (2017) developed an improved isolation protocol and found that 6.7% of the bacteria isolated under aerobic conditions from agricultural peat soil could perform all four denitrification steps, and 16% were able to carry out ${\mathrm{NO}}_{2}^{-}$ reduction to NO, which is considered as the defining step of denitrification (Zumft, 1997; Shapleigh, 2013).

Oxygen gives a higher energy yield from respiration than nitrogen oxides, and is the preferred electron acceptor for denitrifiers. This requires strict genetic regulation, allowing the organisms to respire oxygen during oxic conditions, but to sense decreasing oxygen concentrations in time to initiate the denitrification apparatus, thus avoiding being entrapped in anoxia without the possibility to conserve energy (Højberg et al., 1997). In addition, the transition to anaerobic respiration should be balanced in order to avoid accumulation of the toxic intermediates ${\mathrm{NO}}_{2}^{-}$ and NO. The genetic regulation of denitrification is complex and involves over 50 genes, encoding not only the reductases but also transcription factors and assembly pathways (Zumft, 1997). The regulatory networks of denitrification are diverse, and may differ even between closely related organisms (Spiro, 2012), resulting in a range of regulatory phenotypes (Liu et al., 2013; Lycus et al., 2017). So far, this knowledge is based on detailed studies of a limited number of model organisms. Common to these is that their transcriptional regulation of denitrification genes involves proteins belonging to the bacterial superfamily of Crp/Fnr (cAMP receptor protein/fumarate and nitrate reduction regulatory protein). They include, among others, oxygen sensing proteins that act as transcriptional activators of *nar/nap* and *nos*, such as FnrP (in *Paracoccus denitrificans*), ANR (in *Pseudomonas aeruginosa*) and FixK_2_ in *Bradyrhizobium diazoefficiens*; ${\mathrm{NO}}_{3}^{-}$/${\mathrm{NO}}_{2}^{-}$ sensing proteins such as DNR, DnrD, and NarR (in *Pseudomonas aeruginosa, P. stutzeri*, and *P. denitrificans*, respectively) as well as the two-component regulatory systems NarXL; NarQP and FixLJ; and NO-sensing regulators such as NNR (*P. denitrificans*) and DNR (*P. stutzeri*), which control the transcription of genes encoding Nir, Nor, and N2OR (Rodionov et al., 2005; Spiro, 2012; Gaimster et al., 2018).

A consequence of the large variation in genetic potential and in transcriptional regulation found in denitrifiers is that they differ substantially, not only in their denitrification end products (${\mathrm{NO}}_{2}^{-}$, NO, or N_2_O), but also in their transient accumulation of intermediate products (Lycus et al., 2017). The differences may be large even between closely related organisms. For example, some strains of the genus *Thauera* showed a rapid, complete onset of all four denitrification genes in response to anoxia with little or no accumulation of denitrification intermediates (Liu et al., 2013). Other strains in the same genus instead showed a progressive onset where they first transcribed Nar while inhibiting the transcriptional activation of *nir* until all provided ${\mathrm{NO}}_{3}^{-}$ was consumed, resulting in mM accumulation of ${\mathrm{NO}}_{2}^{-}$. The control of NO accumulation also varies, and batch incubations report values from nM to μM in different organisms (Bergaust et al., 2008, 2010; Lycus et al., 2017). Accumulation of N_2_O by bacteria carrying N2OR can also vary by 2–3 orders of magnitude even between closely related strains (Liu et al., 2013).

In complex microbial communities such as soils, which harbor a large diversity of organisms with different denitrification regulatory phenotypes (Lycus et al., 2017), it can be expected that some populations induce the entire set of denitrification reductases in response to an anoxic spell, while others only initiate part of the pathway either in all cells, or only in a fraction of the population. This would mean that different denitrifier populations (and subpopulations) are active at different times after oxygen depletion, and that organisms with an “early onset” may trigger the onset of other denitrifiers through the production of intermediates such as the signaling molecule NO, which initiates transcription of genes encoding Nir and Nor via NNR-like regulators (Zumft, 2002; Spiro, 2017). In an earlier study of the same soil as the one used here, we observed a major peak in *nir* and *nos* transcription activity during the first ca. 5 h of anoxia (Liu et al., 2010). The main goal of the present investigation was to (1) identify the active groups of denitrifiers in a soil and compare this to the total bacterial denitrifier community in the same soil and (2) to investigate whether there was a shift in the composition of actively transcribing populations during the initial phase of an anoxic spell. We incubated intact soil in microcosms as described by Liu et al. (2010), and sequenced the DNA and cDNA amplicons of the *narG, napA, nirS, nirK*, and *nosZ clade I* genes in samples taken during the first 4 h after oxygen depletion. Since the same primer pairs were used for DNA and cDNA, we could identify the active organisms in the targeted groups at different time points. Our approach allowed us to identify shifts in the composition of active groups of denitrifiers during a transcription peak, in response to anoxia.

A specific, methodological problem that we address is the lack of consensus regarding the choice of similarity threshold values for the clustering of functional gene sequences into OTUs (operational taxonomic units). There is currently no “golden standard threshold” to define a taxon (Konstantinidis et al., 2017), although values between 97 and 99.5% similarity are often used for OTUs based on the 16S rRNA gene (Clarridge, 2004). These thresholds are, however, controversial, especially when applying high throughput sequencing providing orders of magnitudes higher sequence coverage (Konstantinidis et al., 2006; Schloss and Westcott, 2011). Threshold values have also been developed for OTU classification of the denitrification genes (Palmer et al., 2009; Depkat-Jakob et al., 2013), by relating sequence divergence of the functional gene to that of the 16S rRNA gene. This could bias the results since the functional genes in strains that share 97% 16S rRNA sequence identity may have much lower sequence similarity (Gaby and Buckley, 2014) and, moreover, since highly homologous sequences for functional genes may originate from distantly related taxa. In the present study we developed a new strategy to define the sequence similarity threshold of OTUs based on the sequence variation of the targeted region of the functional gene and the number of taxa identified through taxonomy classification. Although the OTU thresholds obtained in this way are sample dependent, the method developed to generate these thresholds can potentially become a general strategy and be applied in any amplicon sequencing studies.

## Materials and Methods

### Preparation of Soil Samples, Anaerobic Incubation, and Gas Kinetics

Peat soil was collected from a hay meadow field in a long-term liming experiment established at Fjaler in western Norway in 1976. The soil, classified as a Sapric Histosol (FAO/ISRIC/ISS), contained on average 45% organic C and 2% organic N. Soil pH had been raised to different levels by addition of shell sand (Sognnes et al., 2006; Mørkved et al., 2007). A plot limed to pH 8.0 (800 m^3^ shell sand ha^-1^) was sampled for the present study. The soil was sieved (4 mm mesh) and subjected to a flooding/drainage treatment with a solution containing 2 mM ${\mathrm{NO}}_{3}^{-}$ (equivalent to ca. 36 μmol N) and 10 mM glutamic acid, as described previously (Liu et al., 2010). Glutamate was added since this soil showed near-constant denitrification rates (no growth) and no detectable transcripts of denitrification genes if no C-source was added, suggesting severe nutrient limitation (Liu et al., 2010). Soil samples (20 g fresh weight) were transferred to 125 ml vials, which were sealed with aluminum caps. The headspace was made near anoxic (∼50 ppmv O_2_ in headspace) by repeated evacuation/filling with He for six times and the vials were then placed in a robotized incubation system which monitored O_2_, NO, N_2_O, and N_2_ continuously (Molstad et al., 2007). Samples were taken 1, 2, and 4 h after O_2_ depletion, in line with earlier studies where transcription of the denitrification genes was observed primarily during the first 5 h (Liu et al., 2010). Destructive sampling was performed by snapshot freezing the soil from each vial in liquid nitrogen. Triplicate vials were sampled at each time point. The samples were then stored at -80°C until further analysis (no longer than 1 week before the mRNA extraction). Three replicate vials were left undisturbed and monitored for gas kinetics for 90 h (sampled for gas concentrations every 3 h) using the robotized incubation system described above, during which all available nitrate was reduced to N_2_ (seen as N_2_ reaching a stable plateau after ∼70 h).

### Nucleic Acid Extraction, Barcoded PCR Amplification, and Pyrosequencing

Extraction of nucleic acids was done using phenol/chloroform, followed by nucleic acid purification using commercial kits, as described previously (Liu et al., 2010). Reverse transcription was performed with random hexamer primers using the Masterscript RT-PCR System (5 Prime GmbH, Hamburg, Germany). Triplicate samples were taken for DNA extraction at the start of the experiment, and for RNA extraction after 1, 2, and 4 h of incubation. DNA and RNA extracts of good quality and quantity were obtained from all samples. The ratios of A_260__nm_: A_280__nm_ measured using Nanodrop spectrophotometer were >1.7 for all the samples. The yield of genomic DNA and total RNA were 19.2–24.6 and 7.9–12.4 μg g^-1^ wet weight soil, respectively. The control samples, which were RNA treated with DNase (without performing reverse transcription), were amplified with the universal 16S rRNA gene primers 27f/1492r (Lane, 1991). No bands were visible on the agarose gels, indicating successful removal of genomic DNA. This was further confirmed by qPCR using the DNase treated RNA as template and cDNA with primer pair 27f/518r (targeting 16S rRNA gene sequences). No signal was detected for the RNA (Ct > 34), confirming that the genomic DNA was successfully digested.

A number of primers were tested, which were used in previous studies for amplification of *napA, narG, nirS, nirK, cnorB*, and *nosZ* clades I and II. Primer combinations which resulted in successful amplification are listed in Supplementary Table S1. For *nosZ*, amplicons were only obtained for clade I, from here on referred to as *“nosZ”*. A barcode sequence was added to one primer in order to identify the source of the sample. PCR was performed using a total volume of 25 μl containing 0.5 unit GoTaq polymerase, 1× GoTaq buffer (Promega), 160 μM (each) dNTP, 0.4 μM (each) primer, and 50 ng of DNA/cDNA. All PCR reactions were carried out using the following conditions: an initial denaturation step at 95°C for 5 min, 11 cycles of denaturation at 95°C for 30 s, annealing with a gradient temperature from 52 to 56°C for 40 s, increased by 0.5°C each cycle, and extension at 72°C for 40 s, followed by 24 more cycles consisting of 95°C for 30 s, 54°C for 40 s, 72°C for 40 s, and a final extension step at 72°C for 10 min. The amplification products were examined on a 1% agarose gel in 1× Tris-acetate-EDTA (TAE), and the targeted bands were cut and purified using PCR product purification columns (Promega). Amplicons were obtained for all the genes from all the DNA samples. From the cDNA, amplicons were obtained for the *napA, narG, nirS*, and *nosZ* genes from all samples, except for the *narG* and *nosZ* genes in the cDNA from the 1 h sampling occasion. No amplicons were obtained for the *nirK* and *cnorB* genes from any of the cDNA samples. The concentrations of the different amplicons, after gel-purification, ranged between 8.5∼121.4 ng/μl. Molar concentrations were calculated based on an average base pair molecular weight of 649 g/mol. The amplicons of similar sizes were pooled in equal numbers (measured as moles) and were sent to the W. M. Keck Center for Comparative and Functional Genomics at the University of Illinois at Urbana-Champaign (United States). A shotgun genomic DNA library was prepared from each pooled PCR sample using the Roche GS Rapid Library Prep Kit following the Roche Rapid Library Preparation Manual instructions (Roche Applied Sciences, Indianapolis, IN, United States). Sequencing was performed on a 454 Genome Sequencer beta FLX+ system (350 flow cycles) according to the manufacturer’s instructions (454 Life Sciences, Branford, CT, United States).

### Sequence Filtering and Analysis

The original sequences in FASTA format and the corresponding quality files were extracted from the raw sequence files by using the “sffinfo” tool from the Mothur pipeline (Schloss et al., 2009). Errors from pyrosequencing and PCR amplification, which can dramatically inflate the detected microbial diversity, were removed using the PyroNoise and SeqNoise algorithms from the AmpliconNoise pipeline (Quince et al., 2009, 2011). Chimera sequences were removed using the software Perseus from the same software package.

### Reference Sequences

An on-the-fly database was constructed for each gene by standalone BLAST (Altschul et al., 1990) with pooled sequences from all samples of the same gene against the nucleotide database from GenBank^1^. The retrieved top 20 hits of each query sequences were pooled together, and the duplicated sequences removed. Detailed taxonomic information was retrieved for each GI number with a Perl script using Entrez Programming Utilities (NCBI E-utilities^2^), the sequences identified as unknown organisms were removed from the database. The resulting file, including an “id-to-taxonomy” file and a sequence file, were used for taxonomy assignment in QIIME (Caporaso et al., 2010).

### Operational Taxonomic Units (OTU)

Denoised reads were clustered into OTUs using the UCLUST (Edgar, 2010) software implemented in QIIME pipeline (Caporaso et al., 2010). Representative sequences from each OTU were selected and taxonomy was assigned using the BLAST method with the default settings (Altschul et al., 1990; Camacho et al., 2009). The UCLUST method relies on a sequence identity threshold set by the user. A cluster is defined by a highly abundant sequence variant, and all its neighboring sequences within the set identity threshold are assigned to the same cluster. For clustering of the 16S rRNA genes a threshold of 0.97 (or 0.95 for genus) is common, but for other, less conserved genes, this is most likely too high. To find an appropriate threshold for the genes in this analysis, we used the approach sketched in Figure 2, where the cutoff value (x-axis) is identity threshold value. We changed the cutoff gradually between 0.6 and 1, determined the number of OTUs and assigned each OTU to a genus each time. The number of discovered genera (blue curve) and OTUs (green curve) shows that the number of taxa reaches saturation when the cutoff value increases above a certain value (illustrated by the red line), while the OTU number above this value increases sharply. Choosing a higher cutoff value will generate more OTUs without discovering more taxonomic groups, indicating we are just splitting genera by a finer resolution. Thus, the red vertical line indicates the threshold we used to cluster OTUs (Figure 2).

### Statistical Analysis

The relative abundances of OTUs (% of total number) were subjected to principal component analysis (PCA) to elucidate major variation and covariation patterns in the cDNA and DNA libraries, and to evaluate the reproducibility of the techniques used by comparing the variation among replicate samples. Significant differences in the cDNA and DNA libraries were investigated by permutational analysis of variance (PERMANOVA, Anderson, 2001). Rarefaction analysis was performed to assess the richness of each library and to evaluate the sampling effort. The data matrix showing the relative abundances of each genus was achieved from QIIME pipeline. The relative abundances were standardized and used for PCA analysis. PCA and rarefaction analysis were performed with the software “R”^3^, using package “FactoMineR”^4^ and package “vegan”^5^, respectively.

#### Nucleotide Sequence Accession Numbers

The sequences obtained in the study were deposited in GenBank with the Accession NOs. MH737773–MH740873.

## Results

### Oxygen Availability During Incubation

**FIGURE 1:**
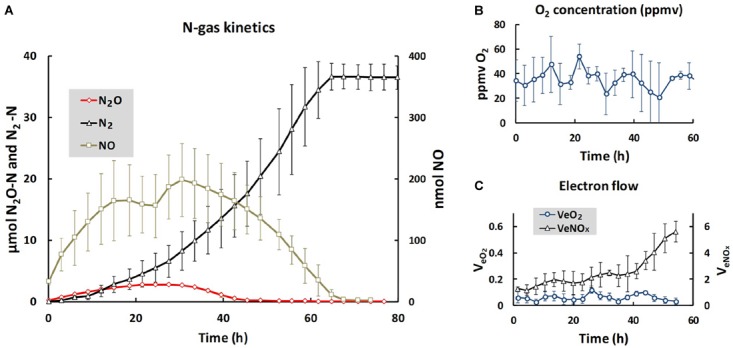
Gas kinetics and electron flow throughout the entire incubation. **(A)** Shows the average amounts of N-gasses per vial: nmol NO (right axis), μmol N_2_O- and N_2_-N (left axis). **(B)** Shows the average O_2_ concentrations (ppmv = uL O_2_ L^-1^), and **(C)** shows the electron flow to terminal oxidases (V_e_O_2_) and to denitrification (V_eNOx_), both as μmol e- vial^-1^ h^-1^. Bars indicate standard deviation (*n* = 3). For N_2_O **(A)**, the standard deviations are too low to be visible. The low and near constant electron flow to terminal oxidases was sustained by the O_2_ leakage through the injection system, which was 50 nmol O_2_ per sampling ( = 16 nmol h^-1^, since the vials were sampled every 3 h). Note that the scale for V_e_O_2_ is one 10th of the scale for V_eNO_X__, thus V_e_O_2_ was approximately 5% of V_eNOx_ during the first 10 h of incubation. Given the fluctuation of oxygen concentrations between 20 and 50 ppmv, the equilibrium concentration of O_2_ in the soil moisture fluctuated between 30 and 75 nM [solubility of oxygen at the incubation temperature (15°C) is 0.0015 mol L^-1^ atm^-1^].

We used the same soil and the same anaerobic incubation procedure as Liu et al. (2010), and obtained essentially identical N gas kinetics throughout the anaerobic incubation (Figure 1): early onset of N_2_ production, transient accumulation of N_2_O peaking after 24 h (2.7 μmol N_2_O-N vial^-1^) declining to zero after 40 h, while the transient NO accumulation (maximum 200 nmol vial^-1^) lasted until nitrogen oxyanions were depleted after 70 h. The cumulated N_2_ reached a plateau at approximately 36 μmol N_2_-N, equivalent to the amount of ${\mathrm{NO}}_{3}^{-}$N in the soil at the start of the incubation (2 mM ${\mathrm{NO}}_{3}^{-}$ added to 20 g soil, and assuming 90% soil moisture). The initial oxygen concentration in the headspace after He washing was 20–50 ppmv (90–220 nmol O_2_ vial^-1^), and fluctuated within this range throughout the entire incubation, despite the input of 50 nmol O_2_ at each gas sampling (16 nmol h^-1^) through leakage during the sampling. Thus, active oxygen consumption took place throughout, although denitrification was the dominant respiratory pathway. For the time increment when the soils were extracted for DNA and RNA (1–4 h after He washing), the electron flow to terminal oxidases accounted for ∼5% of the total respiratory electron flow (Figure 1C). The evidence that the organisms had access to a minimum of oxygen is important, because cells without intact denitrification enzymes need a minimum of aerobic respiration to provide the energy to transcribe denitrification genes and synthesize the enzymes (Hassan et al., 2016).

### OTU Classification

**FIGURE 2:**
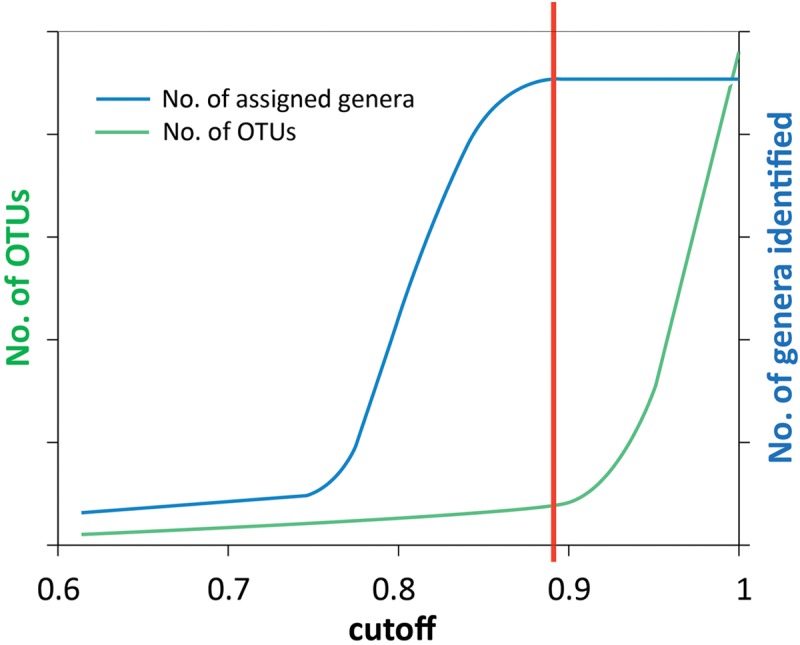
Schematic illustration of how the similarity threshold value for defining OTUs was chosen. The reads were clustered into OTUs at a number of cutoff values ranging from 0.6 to 1. Taxonomy assignment was also performed at each cutoff value. By plotting the number of assigned genera (blue curve) and the number of OTUs (green curve) against the cutoff values, the point where the number of genera is saturated (red line) was chosen as the threshold value to define OTUs. This method was used for all genes analyzed in this study, and the results are shown in Supplementary Figures S1–S6.

Noise removal processes eliminated 35–82% of the bad quality reads. The numbers of sequences before and after noise removal are shown in Table 1. The cutoff values to classify the OTUs for different genes were obtained using the principle described in the M&M section and illustrated in Figure 2. For each gene investigated, a series of sequence similarity threshold values (0.6–1) was selected and the thresholds were generated (shown in Supplementary Figures S1–S6 and listed in Table 1).

**Table 1:** Numbers of amplified sequences, OTUs and assigned genera for the different denitrification genes.

Gene	Total sequence numbers before noise removal	Total sequence numbers after noise removal	OTU threshold (%)	No. of OTUs	No. of assigned genera
*napA*	19140	12358	93	436	34
*narG*	13684	6786	96	1025	70
*nirS*	13286	2389	95	369	30
*nirK*	7665	4178	95	642	23
*qnorB*	10508	4683	83	465	31
*nosZ*	11213	4439	89	164	26


*The OTU thresholds were defined based on the taxonomic assignment, for detailed information see Supplementary Figures S1–S6.*

### Comparison of DNA and cDNA Libraries

**FIGURE 3:**
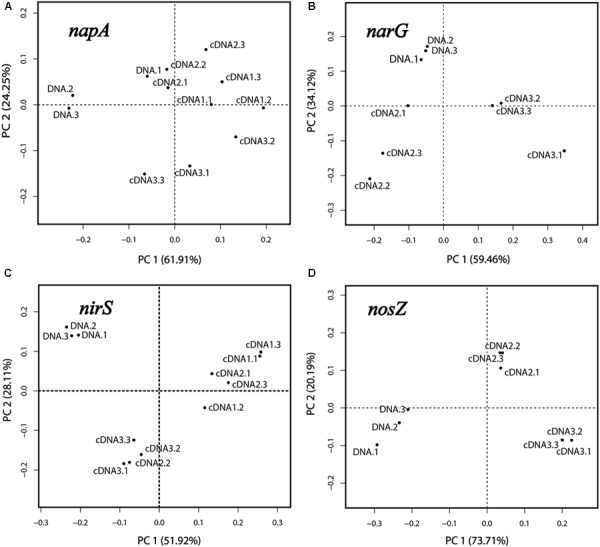
Principal component analysis based on OTUs of the *napA, narG, nirS* and *nosZ* libraries **(A–D)**. Three replicate DNA libraries are shown (designated DNA.1, DNA.2 and DNA.3). For the cDNA, three libraries designated cDNA1, cDNA2, and cDNA3 were obtained, representing the three sampling times (after 1, 2, and 4 h of incubation). For each of these, three replicate samples were analyzed, designated “0.1,” “0.2,” and “0.3”.

Principal component analysis and PERMANOVA analyses were performed for each of the genes *napA, narG, nirS* and *nosZ*, which were the genes for which amplicons were obtained in both the DNA and cDNA samples. For PCA, the relative abundances (in %; standardized values) of OTUs in the respective libraries were used as variables, and the libraries of DNA and cDNA1-3 (sampled at 1, 2, and 4 h) were used as cases. The reproducibility was generally good as seen from the positions of the samples in the PCA plots (Figures 3A–D). For all four genes the DNA libraries were clearly separated from the cDNA libraries along the first principal component (PC1), which explained 51.9–73.7% of the total variation. This reflects the differences in community composition between the total denitrifier community (the DNA library) and the subset of the community that transcribed the same gene (the cDNA library). This was further confirmed by PERMANOVA analysis, which showed that the differences between the DNA and the cDNA for the genes investigated were significant in most cases (Supplementary Table S2). Replicate samples from the cDNA were clustered according to sampling time in the PCA plots (Figures 3A–D), demonstrating a change in the composition of the active part of the microbial community over time, with the 4 h samples clearly separating from the earlier sampling times for all four genes.

### Composition of Total and Active Denitrifier Communities

**FIGURE 4:**
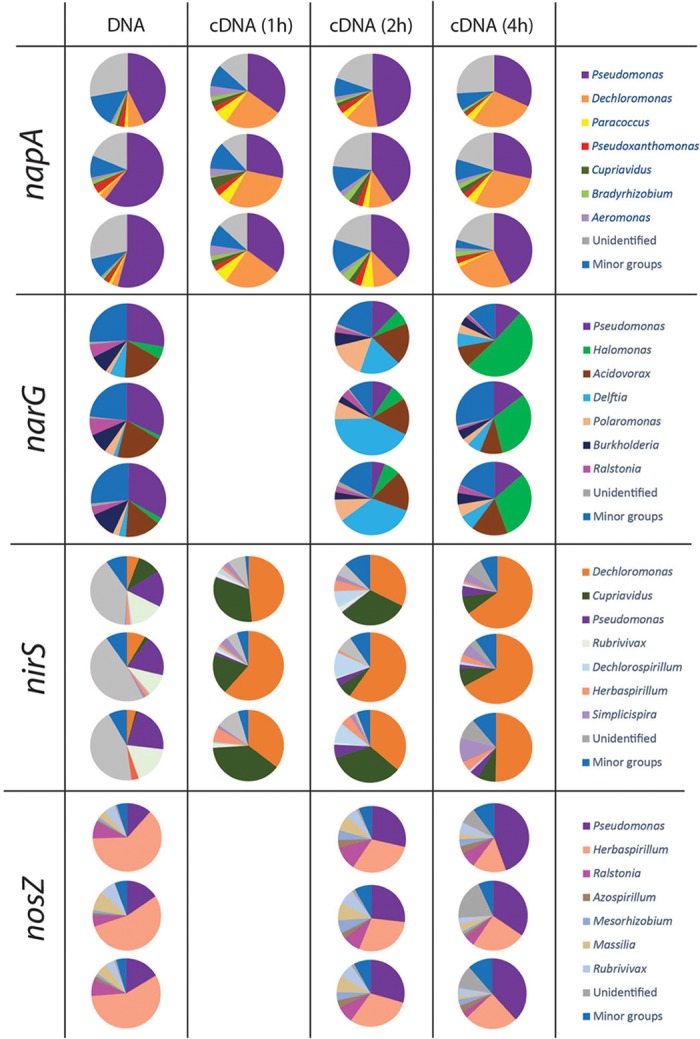
Microbial community structure and shifts in the actively denitrifying populations, revealed by phylogenetic composition analysis of the DNA and cDNA of *narG napA nirS* and *nosZ* genes. The three pie charts in the same box are three replicates. No gene transcription was detected at 1 h for *nosZ* and *narG* genes. The phylogenetic composition of the minor groups is shown in Supplementary File S2.

**FIGURE 5:**
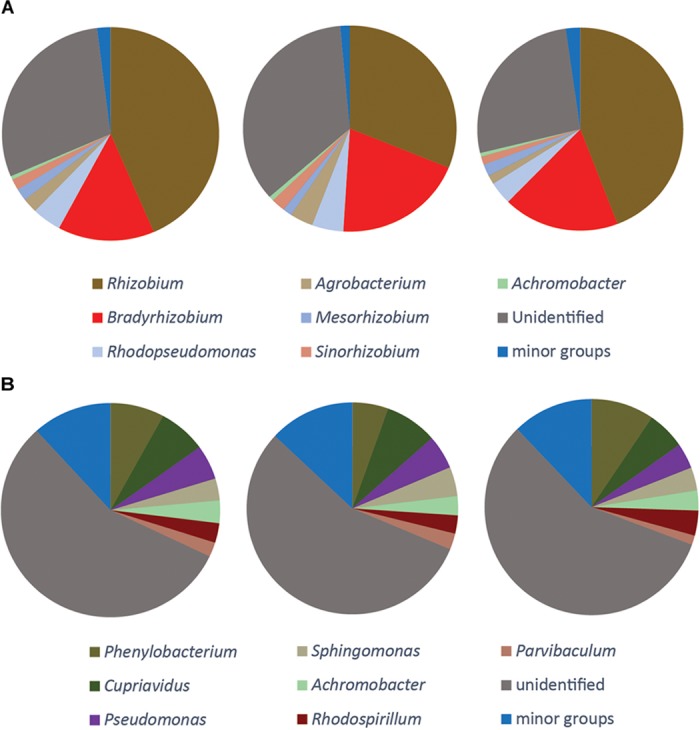
Phylogenetic composition of the DNA of *nirK* gene **(A)**, *qnorB* gene **(B)**. The three pie charts show three replicate samples. No gene transcription was detected for these genes. The seven largest genera are shown and the composition of the minor groups is listed in Supplementary File S2.

Taxonomy was assigned at the genus level and the phylogenetic composition of the seven largest genera for each gene is shown in Figures 4, 5. The minor groups are listed in the Supplementary File S2. For the *narG* gene, which encodes the membrane-bound nitrate reductase NarG (Philippot and Højberg, 1999), amplification products were obtained from the DNA sample and from cDNA sampled at 2 and 4 h, while no amplicons were obtained from the 1 h sampling. A total of 1025 OTUs, assigned to 70 genera, were identified. The most dominant group in the DNA library for this gene was the genus *Pseudomonas*, which accounted for 30.9 ± 2.8% of the total number of sequences. In the cDNA samples, the proportion of this genus was only 9.1 ± 3.1% at 2 h, and increased to 13.2 ± 1.4% at 4 h. The second largest group carrying *narG* was *Acidovorax* (17.8 ± 2.7% in the DNA). This genus accounted for a similar proportion in the cDNA at the 2 h sampling occasion (17.3 ± 1.3%), but decreased to 11.5 ± 3.7% at 4 h. The genus *Delftia*, which only accounted for 3.8 ± 2.5% in the DNA, became the most dominant group in cDNA at 2 h, accounting for 31.6 ± 12.6% of the transcripts. A similar trend was observed for the genus *Halomonas*, which constituted 3.2 ± 2.0% of the DNA community but 38.0 ± 11.4% of the 4 h cDNA community (Figure 4).

Amplicons of *napA* were detected in the DNA as well as in the cDNA from all three sampling occasions. A total of 436 OTUs, assigned to 34 genera, were identified. A considerable portion of the *napA* sequences (25.1 ± 5.4% in the DNA, 20.9 ± 2.1% in the 2 h cDNA and 22.2 ± 3.3% in the 4 h cDNA) had no match in the database and were grouped as “unidentified.” The most dominant group in both DNA and cDNA libraries for this gene was related to the genus *Pseudomonas*, which accounted for 52.3 ± 8.9% in the DNA, 32.4 ± 3.6% in the 1 h cDNA, 42.3 ± 5.1% in the 2 h cDNA and 34.4 ± 7.5% in the 4 h cDNA. A high *napA* gene expression level was observed for the genus *Dechloromonas*, which increased from 4.3 ± 2.4% in the DNA to 25.1 ± 4.7%, 11.5 ± 1.9 and 27.4 ± 2.4% in the 1, 2, and 4 h cDNA samples, respectively.

The *nirS* and *nirK* genes, encoding nitrite reductases, were both detected in the DNA, but only *nirS* was amplified from the cDNA. For the *nirS* gene, a total of 369 OTUs were identified and assigned to 30 genera. As for the other denitrification genes that were transcribed, there were substantial differences between the phylogenetic composition of the amplified genes (in the DNA) compared to that of the transcripts (cDNA) (Figure 4). Organisms belonging to the genus *Dechloromonas* comprised only 5.9 ± 2.1% of the *nirS* sequences in the DNA, but showed very high transcriptional activity of this gene that increased over time, from 48.5 ± 13.1 and 42.7 ± 14.7% of the total sequences in the cDNA at 1 and 2 h, to 61.2 ± 9.2% in the 4 h samples. Another, very active group belonged to the genus *Cupriavidus*, which comprised only 4.4 ± 5.2% of the *nirS* carrying community but 29.7 ± 10.3% of the *nirS* transcripts in the 1 h cDNA samples. In this case the gene transcription levels declined, however, to 23.7 ± 15.2% of the total transcripts at 2 h and to 8.3 ± 0.2% at 4 h. In contrast to the genera *Dechloromonas* and *Cupriavidus, Pseudomonas* was a major genus in the *nirS* carrying community, accounting for 19.3 ± 2.5% of the DNA sequences, but this group of organisms showed very low levels of gene expression, as indicated by the low proportion in the cDNA (<3.5%).

The *nirK* gene was only detected in the DNA samples. The majority of the organisms carrying this gene were different rhizobia, and the genera *Rhizobium, Bradyrhizobium, Mesorhizobium*, and *Sinorhizobium* constituted together more than 60% of all the sequences. Almost one third of the *nirK* sequences (30.3 ± 4.3%) did not match any known phylogenetic neighbors in GenBank (Figure 5A).

Several different primers were tested for the *nor* genes, both for the quinol-oxidizing single-subunit class (*qnorB*) and the cytochrome bc-type complex class (*cnorB*). Of these, successful amplification was only obtained for *qnorB* in DNA samples when using the 2F/7R primers (Supplementary Table S1). A total of 465 OTUs were identified and assigned to 31 genera. More than half of the sequences (56.3 ± 0.7%) were assigned to unidentified groups of organisms. The three most abundant groups were affiliated with the genera *Phenylobacterium, Cupriavidus* and *Pseudomonas*, which comprised 7.7 ± 2.0, 6.9 ± 1.1, and 4.7 ± 0.8% of the total number of sequences, respectively (Figure 5B).

Amplicons of *nosZ* were detected in the DNA and in the 2 and 4 h cDNA samples, but not in the 1 h cDNA samples. In total, 164 OTUs were defined from 4439 sequences, and assigned to 26 genera. The predominant (58.3 ± 4.4%) group in the DNA library belonged to *Herbaspirillum*. This genus comprised a substantial but yet smaller fraction of the cDNA (30.1 ± 0.8 and 22.0 ± 5.5% at 2 and 4 h, respectively). In contrast, the genus *Pseudomonas*, which was a relatively minor fraction of the *nosZ* carrying community with only 14.5 ± 2.6% of the total sequences in the DNA, showed dramatically higher abundance in the cDNA, with 28.3 ± 1.3 and 39.0 ± 5.1% of the total sequences at 2 and 4 h, respectively (Figure 4).

## Discussion

While there is a plethora of studies quantifying denitrification genes in soils, comparably few have determined the transcriptional activities of these genes, and only a few have sequenced the transcripts (Coyotzi et al., 2017; Harter et al., 2017). The present work follows up our earlier studies of the same agricultural peat soil, which demonstrated, both for intact soil and extracted soil microbial communities, that transcription of the *nirS* and *nosZ* genes was initiated as an immediate response to oxygen depletion (Liu et al., 2010). This was seen as a sharp peak in transcript numbers during the first 5 h of incubation, after which transcription dropped below detection limit or stayed at a low level for the rest of the incubation. In the present investigation, we found the same overall pattern and focused on analyzing transcripts in soil samples taken during the first hours of incubation when the peak in transcriptional activity was observed.

The knowledge gathered during the past decades shows substantial similarities in the main elements of the regulatory networks involved in denitrification (Spiro, 2012; Gaimster et al., 2018). Yet, detailed studies of an increasing number of denitrifying organisms have revealed that their regulatory phenotypes are profoundly different (Lycus et al., 2017). Some handle oxygen depletion by a complete onset of all denitrification genes (Liu et al., 2013), which gives rapid access to all available electron acceptors. This is probably an advantage if the anoxia persists, but the production of the entire denitrification machinery comes with a substantial energy investment. It is therefore not surprising to find organisms which express only some of their denitrification genes as a first response to anoxia. This is the case for some *Thauera* strains where the presence of nitrate appears to completely inhibit the transcription of *nirS* (Liu et al., 2013), or in *P. denitrificans* in which only a fraction of the cells in an isogenic population express *nirS* while all cells express *nosZ* and most of them also *narG* (Bergaust et al., 2010; Hassan et al., 2016; Lycus et al., 2018). Such regulatory strategies would translate into a succession of active denitrifiers during the course of an anoxic spell.

Analysis of the DNA showed that, among the dominant genera identified based on the method developed in this study, *Pseudomonas* was the only genus that was detected in all the four gene libraries (Figure 4 and Supplementary File S2). The apparent lack of one or more of the genes in the other genera could be taken to suggest that they have a truncated denitrification pathway (Shapleigh, 2013; Graf et al., 2014), but could just as well-reflect that some of their denitrification genes are not amplified by the primers used. It is well-known that no primers for denitrification genes are universal, and therefore a substantial part of the community remains undetected (Bonilla-Rosso et al., 2016; Decleyre et al., 2016).

Despite the limitations caused by primer biases, the results we obtained from sequencing both DNA and cDNA allowed a direct comparison between the genetic potential and the transcriptional activity of the individual genes. The results show that some groups that dominated in the DNA samples were equally or less abundant in the cDNA, such as *Pseudomonas* (*napA, narG*, and *nirS*), *Acidovorax* (*narG*) and *Herbaspirillum* (*nosZ*). Other groups showed a comparatively high activity with larger relative abundance in the cDNA than in the DNA. These included, e.g., *Dechloromonas* (*napA, nirS*), *Delftia* and *Polaromonas* (*narG*), *Cupriavidus* (*nirS*), and *Pseudomonas* (*nosZ*).

Our approach also allowed a time-resolved dissection of the denitrifying community during the transcription peaks for the individual genes. In accordance with our hypothesis, this revealed shifts in the composition of active groups of denitrifiers (Figure 4). For the genes targeted by the primers used, transcription of *nosZ* was not detected at the first sampling (1 h; Figure 4). Being the last step of denitrification, late transcription of *nosZ* is in itself not surprising. However, since transcription of *nosZ* before other denitrification genes has been shown in *P. denitrificans* (Bergaust et al., 2010), a more likely interpretation is that the *nosZ* gene of such organisms were not targeted by the primers used. Similarly, the lack of early *narG* transcription (Figure 4) only applies to the genes targeted by the present primers. Nitrate reduction most likely took place already after 1 h, providing nitrite for the already active nitrite reductases resulting from *narG* activity in other, non-targeted organisms and/or from *napA* activity. Thus, the results should be interpreted with care; lack of transcripts at a specific time point only reflects lack of transcription of the gene targeted by the primer pairs used. Yet, our approach revealed that the composition of the actively denitrifying community varied over time within the time frame of the 4 h transcription peak for all the genes (Figure 3). Some groups showed an “early onset” of the gene in question (i.e., their transcription started at a higher O_2_ concentration, during O_2_ depletion, compared to groups with a “later onset”) and stayed active throughout, such as *Pseudomonas* (*napA*), *Dechloromonas* (*napA* and *nirS*), and *Cupriavidus* (*nirS*) (Figure 4). The nitrite and NO produced by “early onset” groups will likely act as triggers for the onset of denitrification in other organisms (Zumft, 2002; Spiro, 2017). All groups targeted by the *narG* and *nosZ* primers instead had a later onset. The reason for this is not clear, since transcription of both these genes is controlled by transcriptional regulators belonging to the FNR/CRP family which sense oxygen depletion (Zumft, 1997). The regulation of denitrification is, however, not well-understood and several aspects are probably yet to be unraveled. Recently, it was discovered that in an isogenic population of *Paracoccus denitrificans* only some cells initiated denitrification in response to decreasing oxygen (Lycus et al., 2018), a phenomenon that could explain the observation of late transcription in the present study. For the gene *nirS*, responsible for the defining step of denitrification (reduction of nitrite to a gaseous N-oxide), the genera *Dechlomonas* and *Cupriavidus* comprised a major part of the active community, although transcripts from *Cupriavidu*s became less abundant with time. For *nosZ, Pseudomonas* and *Herbaspirillum* comprised more than half of the detected transcripts, indicating that they play an important role for the reduction of the greenhouse gas N_2_O in this soil.

For the genes *nirK* and *qnor* we could only detect sequences in the DNA, but not in the cDNA samples (Figure 5). The failure to detect *nirK* transcription is consistent with our previous study of the same soil (Liu et al., 2010). Since the gas kinetics (Figure 1) showed complete conversion of ${\mathrm{NO}}_{3}^{-}$ to N_2_, meaning that Nir activity did take place, the ${\mathrm{NO}}_{2}^{-}$ reduction must be attributed to *nirS*-carrying organisms and possibly also to other *nirK* carrying organisms than those targeted by the primers used. In a similar way we did not detect transcription of *qnorB* even though we observed NO reduction, seen as N_2_O production (Figure 1). We tested different published primers and only found one primer pair that successfully amplified *qnor*, but only in the DNA samples. Thus, the observed NO reduction could be from other *qnor* genes, and possibly also from *cnor* genes, from which we could not obtain any amplicons neither from the DNA nor from the cDNA samples. Most studies targeting denitrification genes in complex systems have focused on *nir* and *nos* genes, and it is therefore likely that *nor* genes are less well-represented in the gene databases. This could explain the large portion (56.3%) of unidentified sequences in *qnorB* library in the present study.

A database was constructed for each functional gene based on a blast search of all sets of sequences from the same gene against the NCBI nucleotide database. These databases are dramatically smaller in size than NCBI nucleotide database and therefore facilitated the downstream OTU clustering and taxonomy assignment by reducing the database searching time. The databases were produced based on the sequences generated in the present study, and may not be suitable for analysis of other sequence data sets. Yet, the principle of this strategy could be applied in other studies. Making databases useful for a wider user community would need further optimization, including a general purpose database and related OTU classification tools. A pipeline for this purpose named GENTAX is under development by our group.

To conclude, exposing a soil to near-anoxia induced a sequential transcription of the denitrifying genes, which reflect the presence of a variety of regulatory phenotypes, and an interaction between these via early production of intermediate products (${\mathrm{NO}}_{2}^{-}$ and NO) triggering the transcription in other denitrifiers. The genera *Pseudomonas, Dechloromonas*, and *Herbaspirillum* were identified as dominant, active denitrifiers in this soil, under the given conditions. The results underscore the necessity to perform successive samplings from a number of time points during a short anoxic spell, in order to detect key denitrifiers.

## Author Contributions

ÅF, LB, and BL conceived and designed the experiments. BL and LS developed the OTU clustering strategy. BL and XZ performed the experiments. All authors participated in the writing and improving of the paper.

## Conflict of Interest Statement

The authors declare that the research was conducted in the absence of any commercial or financial relationships that could be construed as a potential conflict of interest.

## Footnotes

1 ftp://ftp.ncbi.nlm.nih.gov/blast/db/

2 http://www.ncbi.nlm.nih.gov/books/NBK25500/

3 http://www.r-project.org

4 http://factominer.free.fr

5 http://vegan.r-forge.r-project.org/
