# Supplementary material for: Rapid Succession of Actively Transcribing Denitrifier Populations in Agricultural Soil During an Anoxic Spell
Source: Front Microbiol. 2019 Jan 8;9:3208. doi: 10.3389/fmicb.2018.03208 (PMC6331397; doi:10.3389/fmicb.2018.03208)
Supplement: Supplementary file 1 [file Data_Sheet_1.docx]

Supplementary Materials

**Table S1.** Primers targeting the functional genes that were analyzed in this study.

| Target gene | Primer name | Length (bp) | reference |
| --- | --- | --- | --- |
| *napA* | V17m, napA4r | 152 | ([Bru et al. 2007](#_ENREF_6)) |
| *narG* | narG-f, narG-r | 173 | ([Bru, Sarr and Philippot 2007](#_ENREF_6)) |
| *nirK* | 1F,5R | 514 | ([Braker et al. 1998](#_ENREF_4)) |
| *nirS* | cd3aF, R3cd | 425 | ([Throback et al. 2004](#_ENREF_36)) |
| *nosZ* | ZF,1622R | 453 | ([Kloos et al. 2001](#_ENREF_17), [Throback, Enwall, Jarvis and Hallin 2004](#_ENREF_36)) |
| *qnorB* | 2F,7R | 637 | ([Braker and Tiedje 2003](#_ENREF_5)) |

**Table S2.** PERMANOVA results based on Bray-Curtis distances using the relative abundance of OTUs from DNA and cDNA libraries. *P* indicates significance level. Values at *P*<0.05 are shown in bold.

|  | *P* value | | | | |  |
| --- | --- | --- | --- | --- | --- | --- |
|  | *napA* | *narG* | | *nirS* | *nosZ* | |
| DNA$\times$cDNA1 | **0.004** |  | **0.002** | |  | |
| DNA$\times$cDNA2 | 0.071 | **0.005** | **0.007** | | **0.001** | |
| DNA$\times$cDNA3 | **0.04** | **0.006** | **0.001** | | **0.001** |  |
| cDNA1$\times$cDNA2 | 0.082 |  | 0.161 | |  |  |
| cDNA1$\times$cDNA3 | 0.074 |  | **0.006** | |  |  |
| cDNA2$\times$cDNA3 | 0.055 | **0.026** | 0.074 | | **0.003** |  |


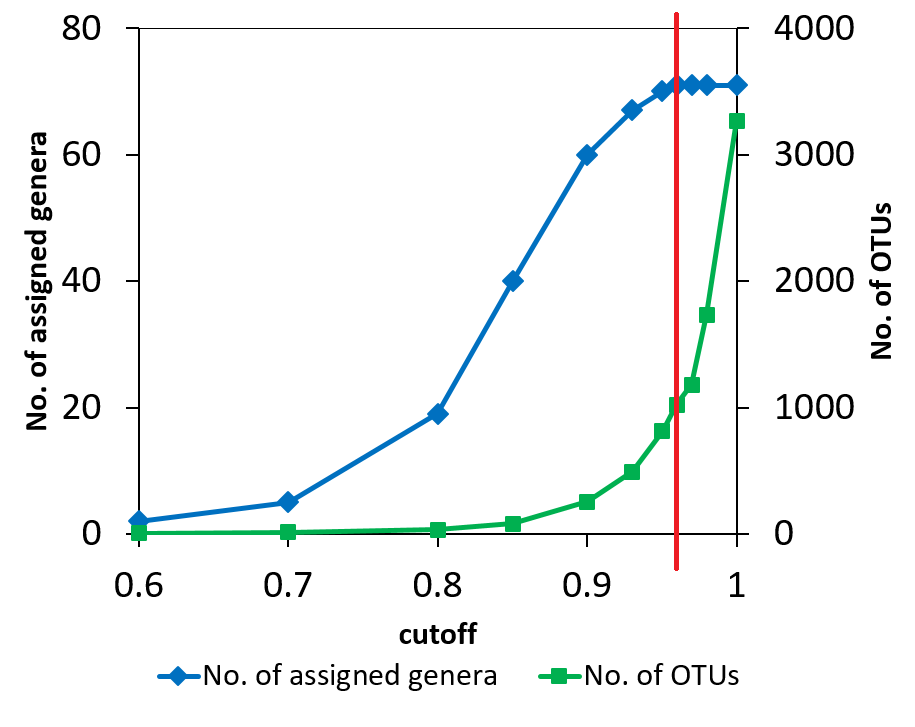


**Figure S1.** Number of OTUs and the corresponding number of assigned genera for *narG*. The sequences were from all DNA and cDNA libraries of the *narG* gene (each with 3 replicates). The OTUs were defined after applying a series of threshold values, and taxonomy was assigned using Qiime. No additional genus was discovered when the cutoff value was greater than 96 %, and this was therefore used as the threshold to define OTUs for this gene.


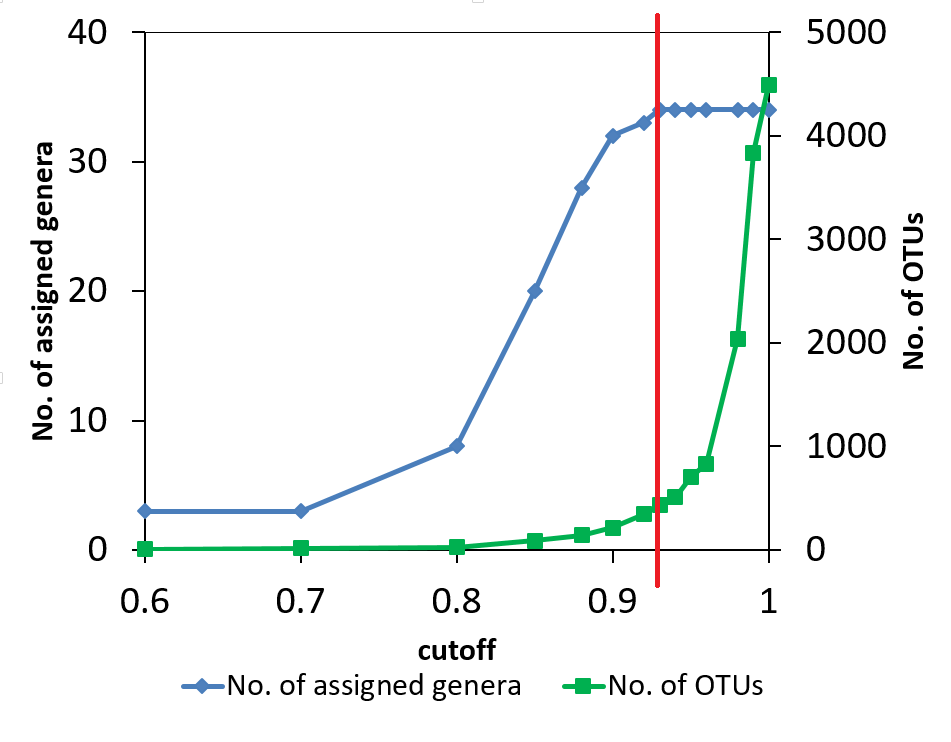


**Figure S2.** Number of OTUs and the corresponding number of assigned genera for the *napA* gene, see legend to Figure S1 for a detailed explanation. A sequence similarity of 93 % was chosen as threshold value to define OTUs for this gene.


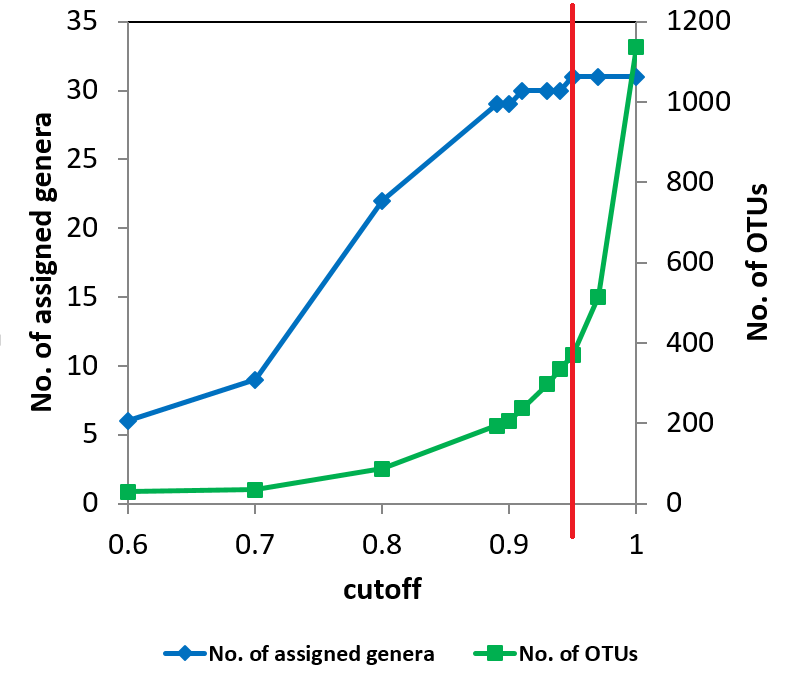


**Figure S3.** Number of OTUs and the corresponding number of assigned genera for the *nirS* gene, see legend to Figure S1 for a detailed explanation. A sequence similarity of 95 % was chosen as threshold value to define OTUs for this gene.


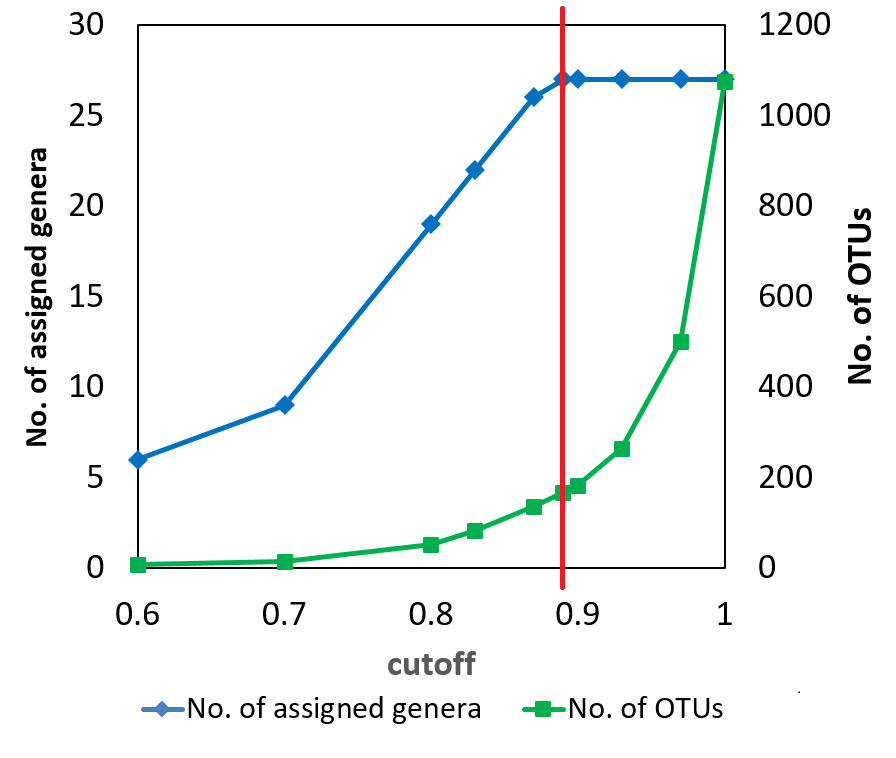


**Figure S4.** Number of OTUs and the corresponding number of assigned genera for the *nosZ* gene, see legend to Figure S1 for a detailed explanation. A sequence similarity of 89 % was chosen as threshold value to define OTUs for this gene.


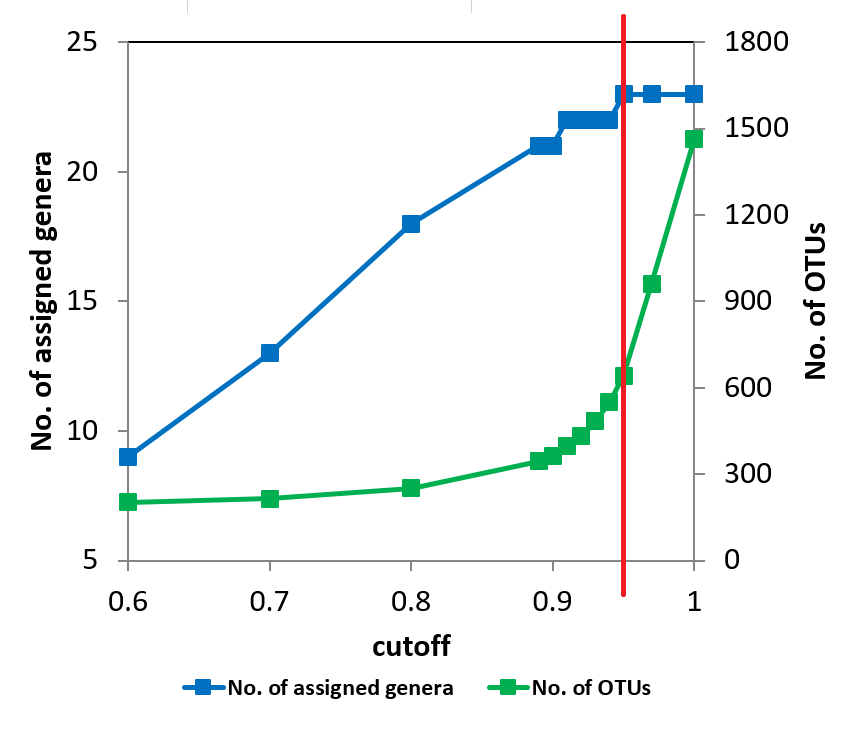


**Figure S5.** Number of OTUs and the corresponding number of assigned genera for the *nirK* gene, see legend to Figure S1 for a detailed explanation. A sequence similarity of 95 % was chosen as threshold value to define OTUs for this gene. Note that only DNA sequences were used in this case since no gene transcription was detected for this gene.


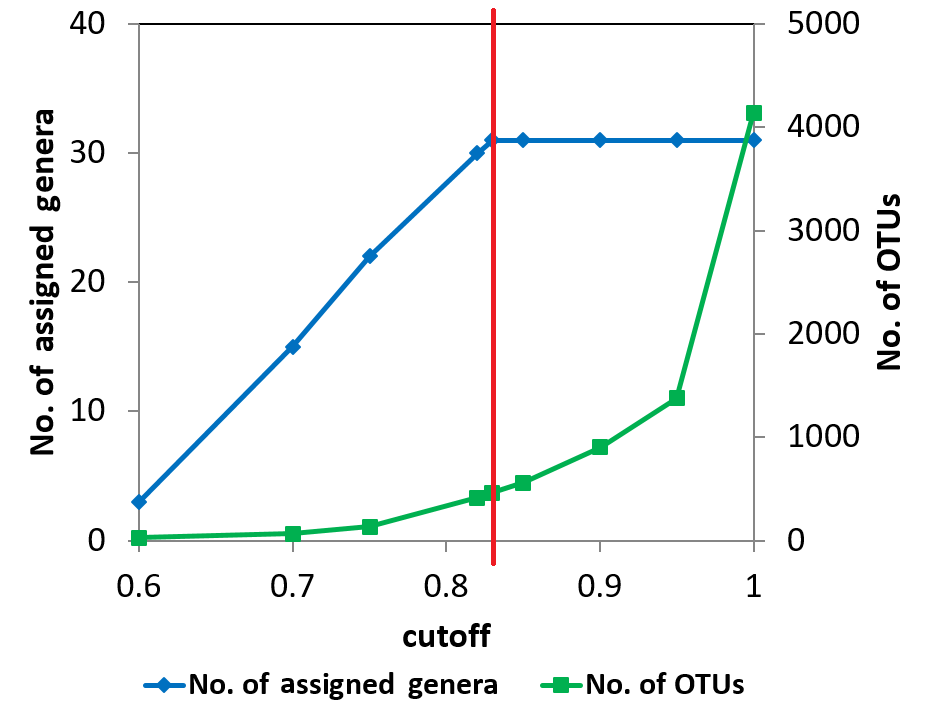


**Figure S6.** Number of OTUs and the corresponding number of assigned genera for the *qnorB* gene, see legend to Figure S1 for a detailed explanation. A sequence similarity of 83 % was chosen as threshold value to define OTUs for this gene. Note that only DNA sequences were used in this case since no gene transcription was detected for this gene.
